# *Grifola frondosa* Polysaccharide F2 Ameliorates Disordered Glucose and Lipid Metabolism in Prediabetic Mice by Modulating Bile Acids

**DOI:** 10.3390/foods14060955

**Published:** 2025-03-11

**Authors:** Ruifang Zhang, Chun Xiao, Tianqiao Yong, Longhua Huang, Huiping Hu, Yizhen Xie, Qingping Wu

**Affiliations:** 1 Guangdong Provincial Key Laboratory of Microbial Safety and Health, State Key Laboratory of Applied Microbiology Southern China, Guangdong Academy of Science Institute of Microbiology, National Health Commission Science and Technology Innovation Platform for Nutrition and Safety of Microbial Food, Guangzhou 510070, China; 15829728206@163.com (R.Z.); hhp201@126.com (H.H.); 2 Guangdong Yuewei Edible Fungi Technology Co., Ltd., Guangzhou 510663, China

**Keywords:** prediabetes, polysaccharides, glucose and lipid metabolism, metabolomics, gut microbiota

## Abstract

Prediabetes (pre-DM) is the buffer period before developing overt type 2 diabetes (T2DM), and the search for novel food agents to protect against pre-DM is in high demand. Our team previously reported that the *Grifola frondosa* (maitake mushroom) polysaccharide F2 reduced insulin resistance in T2DM rats induced by streptozocin (STZ) combined with a high-fat diet (HFD). This study aimed to evaluate the effects of *G. frondosa* polysaccharide F2 on disordered lipid and glucose metabolism and to investigate its mechanisms in pre-DM mice. F2 (30 and 60 mg/kg/d) was administered (i.g.) for 5 weeks to pre-DM mice. The results showed that F2 decreased the fasting blood glucose and lipid profile index of pre-DM mice (*p* < 0.05 or 0.0001). An untargeted metabolomics analysis of feces from pre-DM mice showed that F2 reduced the content of conjugated bile acids, including taurochenodeoxycholic acid and taurocholic acid, and increased the free bile acids of lithocholic acid. The results of 16S rDNA sequencing of feces from pre-DM mice showed that bile salt hydrolase (BSH)-producing bacteria, including *Bacillus*, *Bifidobacterium*, and *Lactococcus*, may be the therapy targets of F2 in pre-DM mice. Through the integrated analysis of untargeted metabolomics and 16S rDNA sequencing, it was found that F2 may ameliorate glucose and lipid metabolism disorders by promoting bile acid metabolism while regulating the abundance of BSH-producing bacteria (*Lactococcus* spp.), suggesting its potential as a functional food ingredient for the prevention of T2DM.

## 1. Introduction

Prediabetes (pre-DM) is the buffer period before the development of overt type 2 diabetes (T2DM), where the fasting blood glucose (FBG) is higher than that of normal controls but lower than that of the diagnostic criteria for T2DM [1]. Pre-DM is mainly characterized by impaired glucose tolerance (IGT) and/or impaired fasting glucose (IFG) [2] as well as intestinal flora disorders [3]. Among them, around 10% of pre-DM cases transition into T2DM [4,5], creating a significant burden for families and countries worldwide. Thus, novel agents that can prevent the transition from pre-DM into T2DM are in high demand.

It is well known that the gut microbiota plays a significant role in the development of pre-DM in patients [6]. In recent years, a substantial body of research has provided evidence for a relationship between the gut microbiota and systemic glucose metabolism [7]. The gut microbiota degrades polysaccharides that cannot be directly utilized by the body by secreting various enzymes, so polysaccharides with different structures shape different gut microbiota. Polysaccharides can act as key modulators and as prebiotics, promote the production of gut hormones, or change bile acid profiles, resulting in the improvement of insulin sensitivity and reversing IG/IFG in pre-DM patients by modulating the gut microbiota [8,9,10].

*Grifola frondosa*, generally known as hui-shu-hua in Chinese and maitake in Japanese, is an edible and medicinal mushroom with bioactivities such as antioxidant activity, immune regulation, and hypoglycemic effects [11]. The main active components in *G. frondosa* are polysaccharides, which are the components of the cell wall of the fruiting body of *G. frondosa*, mainly composed of α-1,4, β-1,4, β-1,6, and β-1,3 linked heteropolysaccharides [12]. Previous studies focused on the regulation of the insulin signaling pathway by *G. frondosa* antidiabetic polysaccharides, such as F2 [13], MT-α-glucan [14,15,16], X peptidoglycan [17,18], SX glycoprotein [19,20], and heteropolysaccharide GFP-N [21].

GFP refers to the crude polysaccharides of the fruiting body of *G. frondosa*. GFP intervention could ameliorate hyperglycemia and hyperlipidemia in type 2 diabetic mice/rats, probably through the modulation of intestinal microflora and the regulation of mRNA expression levels of the genes involved in hepatic glycolipid metabolism [22,23,24].

Chen et al. [21] reported that a novel *G. frondosa* polysaccharide, GFP-N, with a molecular weight (Mw) of 1.26 × 10^7^ Da, decreased fasting blood glucose levels, alleviated hepatic insulin resistance, and regulated the intestinal bacterial structure (increasing the species abundance of *Akkermansia*, *Lactobacillus*, and *Turicibacter*) in type 2 diabetic mice. Xiao et al. [25] reported that *G. frondosa* GF5000 (Mw > 5000 Da) improved glucose metabolism and insulin resistance by alleviating inflammation and modulating the composition of the gut microbiota, increasing the abundance of *Lactobacillus*, *Turicibacter*, and *Streptococcus* and decreasing that of *Prevotella* and *Bifidobacterium* in HFD- and alloxan-induced diabetic rats.

However, the mechanisms underlying the improvements in glucose and lipid metabolism by *G. frondosa* polysaccharides and their regulation of the gut microbiota structure remain unclear. Combining 16S rDNA gut microbiome sequencing with metabolomics is considered a reliable method for analyzing structural changes in the gut microbiota and the interaction mechanism between the gut microbiota and its host [26]. Here, we aimed to evaluate the effects of *G. frondosa* polysaccharide F2 on disordered lipid and glucose metabolism and to investigate its mechanisms in pre-DM mice. These research results can be applied to the development of new antidiabetic functional foods and health products.

## 2. Materials and Methods

### 2.1. Reagents

Ethanol (≥99.7%) was supplied by Guangzhou Chemical Reagent Factory (Guangzhou, China). NaCl (≥99.0%) and NaOH (purity ≥ 96.0%) were obtained from Sangon Biotech Co., Ltd. (Shanghai, China). Methanol and acetonitrile (chromatographic grade) were purchased from Merck (Shanghai, China). Ultra-pure water was obtained by filtering tap water through a Millipore S.A.S. 67120 water purification system (Millipore Co., Molsheim, France). Formic acid and ammonium acetate were purchased from Merck Co. (Shanghai, China).

### 2.2. Preparation of G. frondosa Polysaccharide F2

The dried fruiting bodies of *G. frondosa* were provided and identified by Guangdong Yuewei Edible Fungus Technology Co., Ltd. (Guangzhou, China). They were grown in Huangtian Town (north latitude 27°25′~27°51′, east longitude 118°50′~119°30′), Qingyuan County, Lishui City, Zhejiang Province, China, in spring 2020.

The extraction and purification of *G. frondosa* polysaccharide F2 in this research were consistent with our previous reports [13]; that is, the crude polysaccharide, extracted with water and precipitated with alcohol, was applied to DEAE-Sepharose Fast Flow and washed with 0.1 M NaCl. The F2 was dialyzed and freeze-dried for animal tests.

### 2.3. Animals and Drug Administration

All animal experiments (approval ID: GT-IACUC202009105) were approved by the Animal Care and Use Committee of Institute of Microbiology, Guangdong Academy of Science (Guangzhou, China). Four-week-old male BALB/c mice (about 18 g) were purchased from Beijing HFK Bioscience Co., Ltd. (Beijing, China) and then kept at 22–23 °C under a relative humidity of 56 ± 10% and a light–dark cycle of 12/12 in the Center of Animal Care and Use of Institute of Microbiology, Guangdong Academy of Science. All animal experiments were approved by the Animal Care and Use Committee of the Institute of Microbiology, Guangdong Academy of Science (Guangzhou, China). The ethical review board of the Animal Care and Use Committee provided ethical approval (Approval ID: GT-IACUC202009105).

Freeze-dried F2 polysaccharide were dissolved in distilled water and freshly prepared for daily use. After adaption for 1 week, all mice were divided into 5 groups (8 mice/group) and administered gavage (i.g.) as follows: (1) normal control (NC, sterile water), (2) pre-DM control (DC, sterile water), (3) positive control (PC, metformin, 100 mg/kg/d), (4) F2 group at a low dose (F2-L, 30 mg/kg/d), and (5) F2 group at a high dose (F2-H, 60 mg/kg/d). The dosage of F2 administration was adjusted slightly based on our laboratory’s previous animal experiments [13,25]. The NC group was fed a standard normal chow diet, and the other groups were fed a high-fat and high-sugar (HFHS) diet [25] (the ingredients are outlined in Table S1) for 5 weeks. The FBG levels of blood collected from their tail veins were measured after fasting for 5 h once a week with an ACCU-CHEK^®^ blood glucose meter.

In the final week, feces were collected, and an oral glucose tolerance test (OGTT) was performed via gastric gavage with 2 mg/kg D-glucose (Sangon Biotech Co., Ltd., Shanghai, China) following 12 h of fasting according to our previous study [25]. At the end of the study, the mice were fasted and weighed, and then blood samples were collected from the orbital sinus, after which the mice were anesthetized and sacrificed through cervical decapitation. Blood samples were centrifuged at 3500 rpm for 15 min at 4 °C and then stored at −80 °C for further analysis.

### 2.4. Biochemical Parameters

Blood serum total cholesterol (TC), triglyceride (TG), high-density lipoprotein cholesterol (HDL-c), and low-density lipoprotein cholesterol (LDL-c) were determined using commercially available assay kits (Mindray, Shenzhen, China) on an automatic biochemical analyzer (Mindray, BS480, China). Fasting serum insulin (FSI) was determined using an ^125^I-labled insulin radioimmunoassay kit from Beijing Beifang Biotech Institute (Beijing, China). Insulin resistance (HOMA-IR) was calculated as follows:

HOMA-IR = FBG (mmol/L) × FSI (mIU/L)/22.5

### 2.5. UPLC-MS/MS Analysis of Fecal Samples

An extraction solvent (250 μL) consisting of methanol–acetonitrile–ultra-pure water (40%:40%:20%, *v*/*v*/*v*) was added to 50 mg feces samples (8 samples/group, collected from all animals) and then homogenized with a homogenizer (Tissuelyser-24, Shanghai Jingxin Industrial Development Co., Ltd., Shanghai, China) at 45 Hz for 5 min. After ultrasonication for 5 min, the samples were placed in a refrigerator at 4 °C for 1 h, followed by centrifuging at 12,000 rpm for 10 min at 4 °C. The supernatant was vacuum-dried and then re-dissolved in 100 μL of acetonitrile–water (1:1, *v*:*v*), vortexed for 30 s, and centrifuged at 12,000 rpm for 15 min. The resulting supernatant was applied to UPLC-MS/MS (Q-Exactive Plus, Thermo Scientific, Waltham, MA, USA) equipped with a 100 × 2.1 mm 2.6 μm Accucore TM C18 Column (Thermo Scientific, Part No 17126-102130). The temperatures of the injector and column oven were kept constant at 15 °C and 40 °C, respectively. The injection volume was 2 µL and the flow rate was 3 mL/min.

For metabolomics analysis, the original spectrum information was pre-processed using Compounds Discovered 3.1 (Thermo Scientific, Waltham, MA, USA) to export a data matrix with metabolite name, molecular weight, and peak area. The normalized data matrix was imported into SIMCA 14.1 (Umetrics AB, Umea, Sweden) for a multivariate statistical analysis, and then a principal component analysis (PCA) and an orthogonal partial least-squares discrimination analysis (OPLS-DA) were performed. Different expressed metabolites (DEMs) were screened following the criteria of VIP > 1, *p* < 0.05, and fold change > 2 or <0.5. The data matrix of the DEMs was imported into MetaboAnalyst (https://www.metaboanalyst.ca/, accessed on 8 April 2021) to conduct a pathway enrichment analysis. Correlation heatmapping between various bile acids and physiological traits was performed using the OmicStudio tools at https://www.omicstudio.cn, accessed on 8 April 2021.

### 2.6. Fecal Microbiota Composition Analysis [27]

DNA was isolated from frozen stool samples using a commercial DNA extraction kit (HiPure Stool DNA Kit DNA, MgBio, Shanghai, China) and quantified using a Qubit 3.0 fluorometer (Equalbit 1 × dsDNA HS Assay Kit, Vazyme, Nanjing, China). Then, the 16S rDNA gene (V3-V4) region of the DNA was amplified using primers as follows: forward, 5′-CCTACGGRRBGCASCAGKVRVGAAT-3′; reverse, 5′-GGACTACNVGGGTWTCTAATCC-3′. Polymerase chain reaction (PCR) products were detected using 1.5% agarose gel electrophoresis (C1000 Touch Thermalcycler, BioRad, Hercules, CA, USA). The DNA library concentration was validated using a Qubit 3.0 fluorometer. After quantifying the library to 10 nM, the samples were loaded on an Illumina NovaSeq instrument (Illumina, San Diego, CA, USA) according to the manufacturer’s instructions for sequencing and analysis.

After raw fastq files were demultiplexed and quality filtered, and a taxonomy analysis of operational taxonomy units (OTUs) was performed at each taxonomical level (phylum, class, order, family, and genus) with QIIME (version 1.9.1). The beta diversity of the samples was evaluated using a principal coordinate analysis (PCoA) based on the Bray–Curtis distance. To identify the specific bacterial taxa at the genus, family, order, class, and phylum levels in every group, a taxonomic cladogram was derived from the linear discriminant analysis effect size (LEfSe).

### 2.7. Correlation Analysis

Correlation heatmaps of bile salt hydrolase (BSH)-producing bacteria and physiological traits, BSH-producing bacteria, and bile acids were generated using the OmicStudio tools at https://www.omicstudio.cn, accessed on 12 April 2021.

### 2.8. Statistical Analysis

All data are presented as means ± standard deviations (SDs). Differences between groups were determined using one-way ANOVA analysis followed by Tukey’s multiple comparison test. Statistical differences were considered significant at *p* < 0.05, 0.01, 0.001, and 0.0001 levels. GraphPad Prism software version 19 was used for all analyses.

## 3. Results

### 3.1. F2 Ameliorated Lipid and Glucose Metabolism in Pre-DM Mice

On the fourth and fifth weeks, the FBG of the DC was markedly elevated in comparison with the NC (*p* < 0.01 and 0.0001, Table 1), and metformin showed stable and potential efficacy in lowering FBG (*p* < 0.05 and 0.01) in comparison with the DC. It is worth noting that F2-L at 30 mg/kg/d lowered the FBG in pre-DM mice in the second week, and F2-L at 30 mg/kg/d and F2-H at 60 mg/kg/d showed a remarkable hypoglycemic effect (*p* < 0.05, 0.01, and 0.0001) on the fourth and fifth weeks in comparison with the DC. Specially, F2 at 30 mg/kg/d and 60 mg/kg/d decreased the FBG of pre-DM mice (7.34 ± 0.67 mmol/L) to 6.12 ± 0.55 mmol/L and 6.37 ± 0.57 mmol/L (*p* < 0.05 or 0.0001), showing excellent hypoglycemic effects of approximately 13–17%. Overall, F2 ameliorated glucose metabolism disorders in pre-DM mice.

The OGTT results (Table 2) revealed that the DC had a higher blood glucose at 1.5 h and 2 h (6.77 ± 1.43 and 5.94 ± 0.89 mmol/L) than the NC (5.07 ± 0.36 and 4.58 ± 0.39 mmol/L, *p* < 0.0001), demonstrating a significant impaired glucose tolerance (IGT), which is a typical character of pre-DM. However, metformin showed lower glucose levels of 5.54 ± 0.84 and 4.83 ± 0.71 mmol/L at 1.5 h and 2 h than the DC, indicating the amelioration of IGT in metformin-treated pre-DM mice. F2 at the two doses exhibited glucose levels at 6.31 ± 0.65 and 5.51 ± 0.70 mmol/L at 1.5 h and 2 h, which were only slightly lower than those of DC.

**Figure 1:**
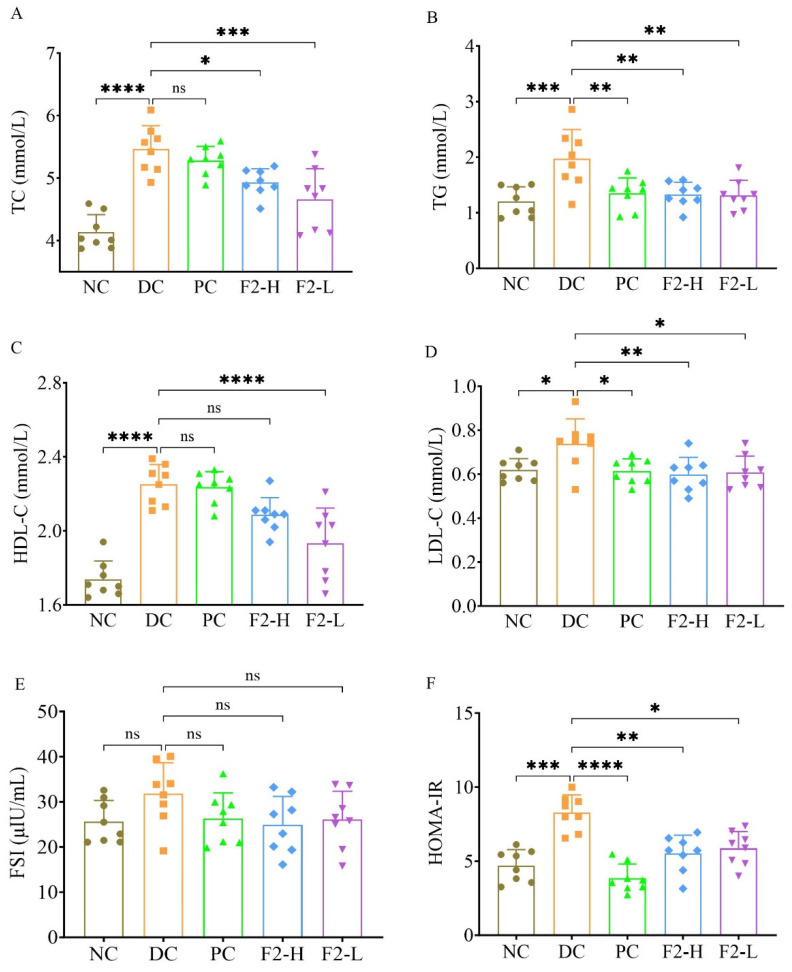
F2 ameliorated lipid and glucose metabolism in pre-DM mice: (**A**) TC; (**B**) TG; (**C**) LDL-C; (**D**) HDL-C; (**E**) FSI; (**F**) HOMA-IR. Values are represented as mean ± SD. n = 8. * *p* < 0.05, ** *p* < 0.01, *** *p* < 0.001, **** *p* < 0.0001, ns = no significance vs. DC.

TC, TG, LDL-C, and HDL-C were significantly increased in the DC in comparison with the NC (*p* < 0.0001, 0.001, or 0.05, Figure 1A–D). As for TG and LDL-C, metformin showed a significant decrease in TG and LDL-C compared with the DC (*p* < 0.01 or 0.05). However, compared with the DC, F2-L had lower levels of TC, TG, LDL-C, and HDL-C (*p* < 0.0001, 0.01, or 0.05), and F2-H decreased the levels of TC, TG, and LDL-C (*p* < 0.05 or 0.01). Overall, F2 demonstrated an amelioration effect in lipid metabolism against pre-DM in mice.

No significant difference was observed in fasting serum insulin (FSI) among all groups (Figure 1E). In terms of HOMA-IR, it was significantly increased (*p* < 0.001) in the DC group compared with the NC group. Both F2-H and F2-L significantly decreased HOMA-IR (*p* < 0.01 or 0.05, Figure 1F), suggesting an improvement in insulin resistance.

### 3.2. Hypoglycemic Effect of F2 Featured with Changed Bile Acids

#### 3.2.1. Principal Component Analysis (PCA) for Metabolomics

**Figure 2:**
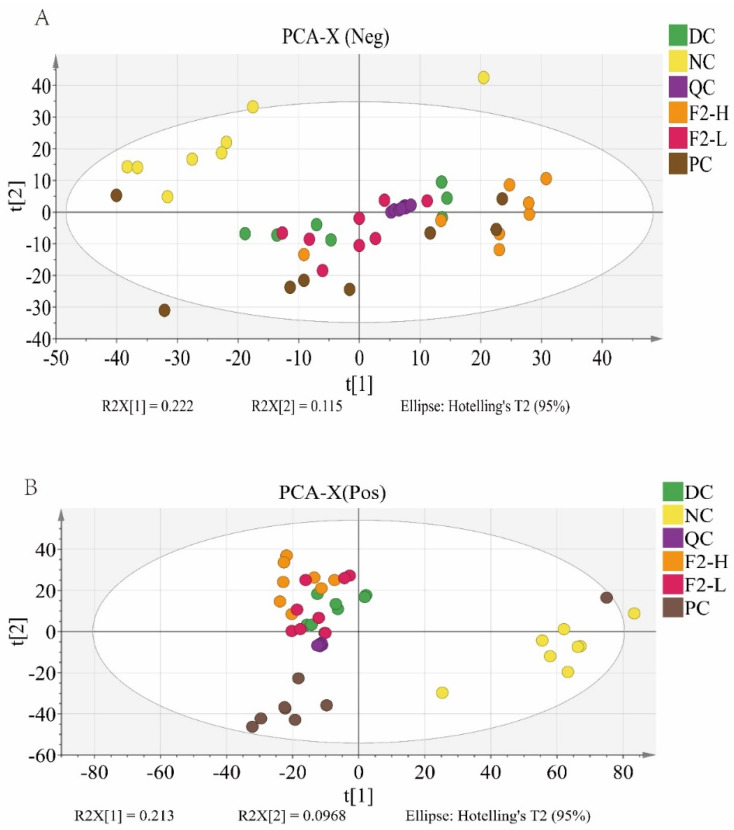
PCA score plots of the (**A**) positive model and (**B**) negative model of the fecal metabolomics of the pre-DM mice treated with F2. n = 8.

The PCA was first performed to examine the metabolic distinction among the groups of interest. On the negative and positive model (Figure 2A,B), the quality control (QC) samples overlapped, demonstrating that the data quality was stable and reliable. Furthermore, clear separations were observed among the NC, DC, F2-L, and F2-H groups, showing distinctive metabolic features.

#### 3.2.2. The Characteristics of Metabolic Disorder in Pre-DM Mice

A total of 70 DEMs were screened out in the comparison of the DC vs. NC. It is worth noting that these DEMs included bile acids such as deoxycholic acid (DCA), glycocholic acid (GCA), lithocholic acid (LCA), and cholic acid (CA); amino acids such as taurine and pipecolic acid; lysophosphatidylcholines such as LPC 18:2, LPC 20:4, LPC 14:0, and LPC18:3; and long-chain fatty acids such as linoleic acid, palmitoleic acid, hypogenic acid, and adrenic acid (Table S2).

**Figure 3:**
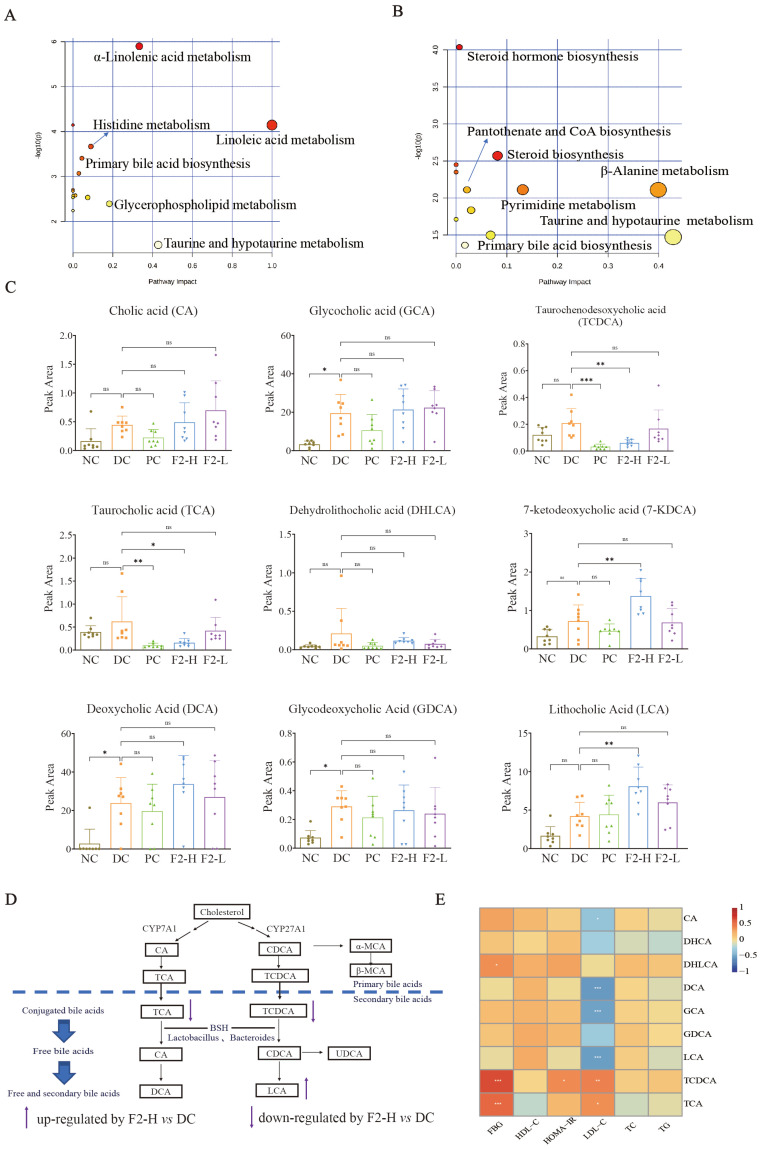
Hypoglycemic mechanism of F2 featured changed bile acids in pre-DM mice: (**A**) pathway analysis of NC vs. DC; (**B**) pathway analysis of F2-H vs. DC; (**C**) influence of F2 on nine bile acids in pre-DM mice; (**D**) effect of F2 on bile acid metabolism; (**E**) correlation heatmap between nine bile acids and physiological traits (red represents positive correlation; blue represents negative correlation). Values are represented as mean ± SD. n = 8. * *p* < 0.05, ** *p* < 0.01, *** *p* < 0.001, and ns = no significance.

Table S3 shows that the DEMs in the comparison of the DC vs. NC were mainly enriched in α-linolenic acid metabolism, linoleic acid metabolism, glycerophospholipid metabolism, and taurine and hypotaurine metabolism with impact values above 0.1 and false discovery rates (FDR) less than 0.05 (Figure 3A).

#### 3.2.3. Regulated Bile Acids in Pre-DM Mice May Be the Targets of F2 in the Alleviation of Pre-DM

A total of 37 DEMs were screened out in the comparison of F2 vs. DC (Table S4). In the pathway enrichment analysis of F2-H vs. DC, primary bile acid biosynthesis showed the most hits, and taurine and hypotaurine metabolism presented the highest impact factor (Figure 3B and Table S5).

Figure 3C shows the peak areas of the nine bile acids in each experiment. Glycocholic acid (GCA), deoxycholic acid (DCA), and glycodeoxycholic acid (GDCA) increased significantly in the DC group compared with NC (*p* < 0.05), suggesting that disturbed lipid metabolism in pre-DM mice may be attributed to the changes in bile acid metabolism. F2-H intervention significantly decreased the content of taurochenodeoxycholic acid (TCDCA) (*p* < 0.01) and taurocholic acid (TCA) (*p* < 0.05) and significantly increased 7-ketodeoxycholic acid (7-KDCA) and LCA (*p* < 0.01) compared with DC.

In summary, F2 reduced the content of conjugated bile acids, including TCDCA and TCA, and increased the free bile acids of LCA. These findings indicate that F2 may effectively regulate bile acid metabolism in pre-DM mice (Figure 3D).

#### 3.2.4. Correlation Between Bile Acids and Physiological Traits

A Spearman rank correlation analysis of the bile acids and lipid and glucose physiological traits (Figure 3E) revealed that TCA and TCDCA had a strongly positive relationship with FBG and LDL-C, while DCA, GCA, and LCA were found to be significantly negatively correlated with LDL-C. Notable decreases in TCA and TCDCA along with a significant increase in LCA in the F2-H group (Figure 3C) suggests that F2 may ameliorate lipid and glucose metabolism by modulating bile acids in pre-DM mice, particularly through downregulating TCA and TCDCA and upregulating LCA.

### 3.3. Structural Characteristics of the Fecal Microbiome in Pre-DM Mice and F2-Treated Pre-DM Mice

**Figure 4:**
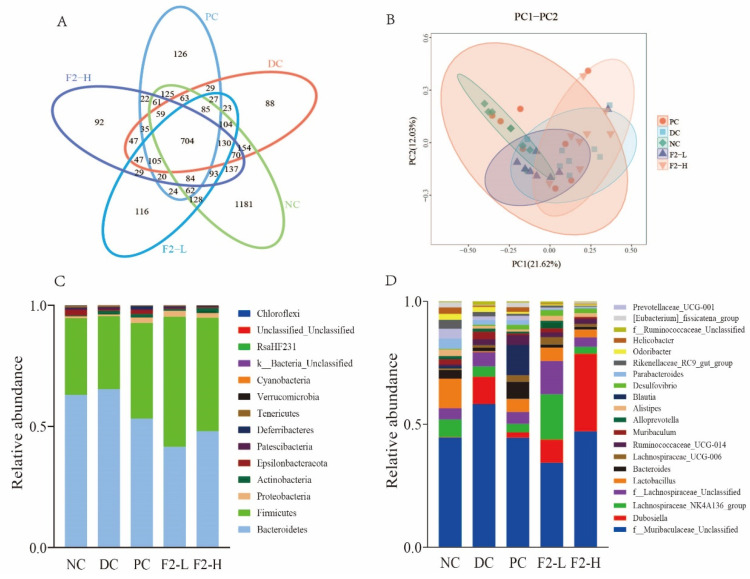
The reshaping effect of F2 on the dysbiosis of the gut microbiota in pre-DM mice induced by HFHS feeding: (**A**) flower plot; (**B**) PCoA plot; (**C**) relative abundance of the bacteria community at the phylum level; (**D**) relative abundance of the bacteria community at the genus level.

The Venn diagram presented in Figure 4A shows that there were 1104 unique OTUs in the NC, 75 unique OTUs in the DC, 122 unique OTUs in the PC, 95 unique OTUs in the F2-L group, and 78 unique OTUs in the F2-H group. Based on the weighted UniFrac distance, the PCoA analysis results showed distinct clustering of the microbiota composition among these groups (Figure 4B). At the phylum level, Bacteroidetes and Firmicutes were dominant in all samples. Increased abundance of Bacteroidetes and decreased abundance of Firmicutes were observed in the DC group compared with the NC group (Figure 4C), while polysaccharide F2 treatment decreased the abundance of Bacteroidetes and increased the abundance of Firmicutes.

At the genus level, the abundance of f__*Muribaculaceae*_Unclassified and *Dubosiella* was increased, whereas the abundance of *Lactobacillus*, *Bacteroides*, and *Lachnospiraceae*_NK4A136_group was decreased in the DC mice (Figure 4D), demonstrating that they may be the markers of the microbiota of pre-DM. At the genus level, the abundance of *Lactobacillus* and *Lachnospiraceae*_NK4A136_group was increased in the F2-L and F2-H groups compared with the DC group, while the abundance of f__*Muribaculaceae*_Unclassified was decreased (Figure 4D).

**Figure 5:**
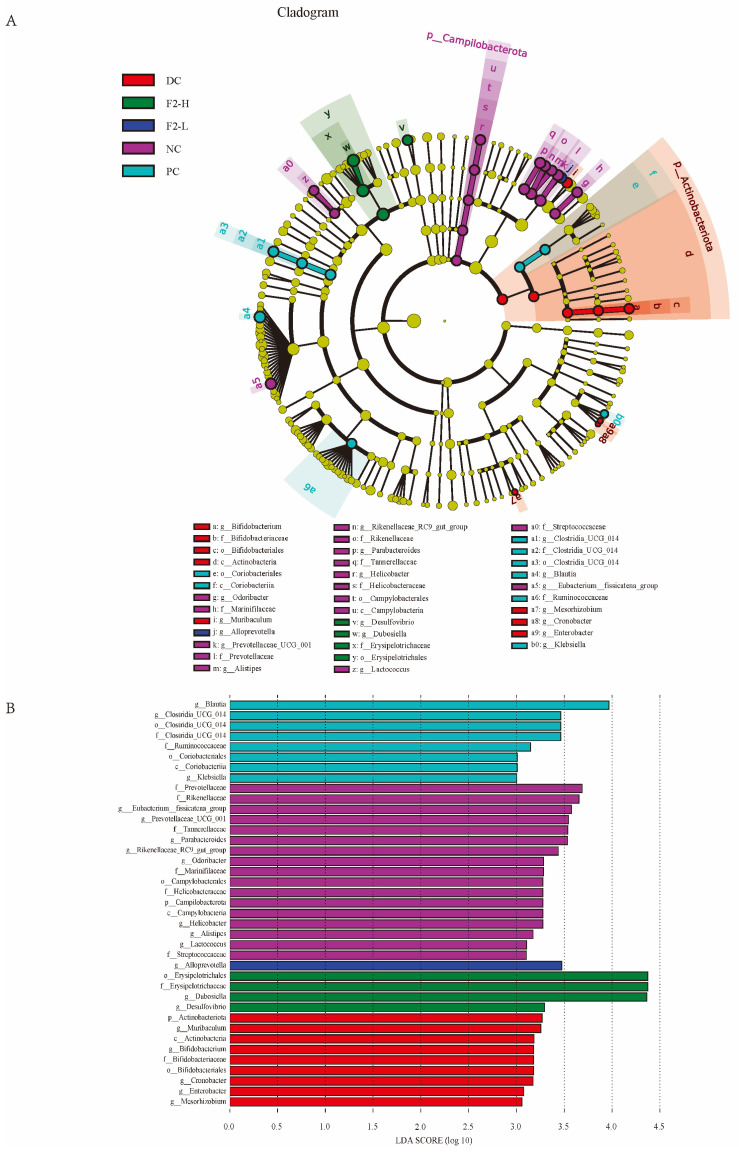
LEfSe (LDA effect size) comparisons of gut microbiota: (**A**) cladogram plot; (**B**) LEfSe results (red = DC; green = F2-H; blue = F2-L; purple = NC; light blue = PC).

LEfSe analysis is usually used to identify differences among multiple groups. At the genus level, the HFHS diet in the DC group impacted the bacterial genera of *Bifidobacterium* spp. and *Muribaculum* spp., which were screened out on the basis of log 10 LDA > 3.0 (Figure 5A,B). The enriched bacteria in the F2-L and F2-H groups were *Alloprevotella* spp., *Dubosiella* spp., [*Eubacterium*]_*xylanophilum*_group spp., and *Desulfovibrio* spp.

### 3.4. Correlations Between Physiological Traits and BSH-Producing Bacteria

**Figure 6:**
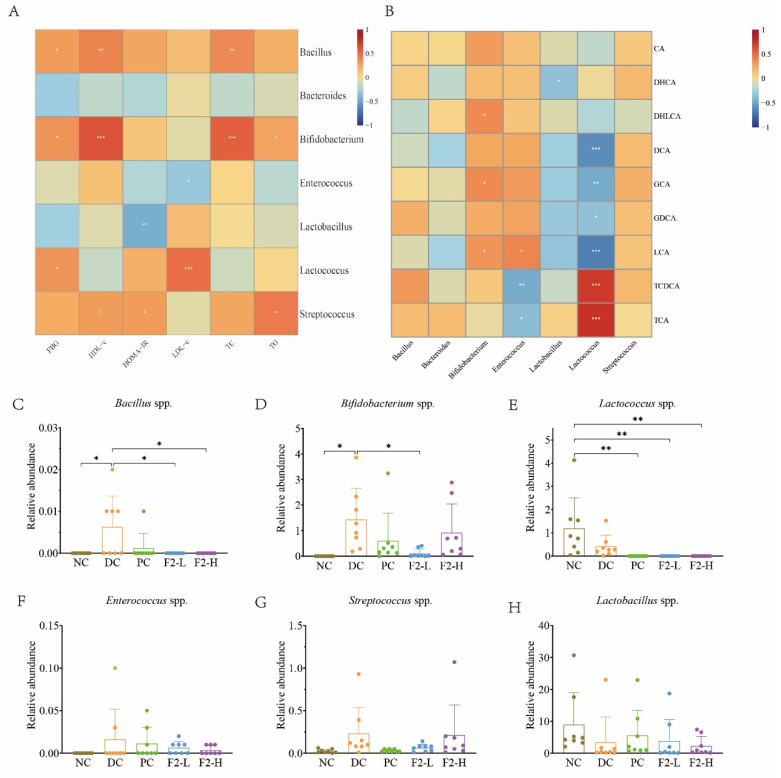
(**A**) Correlation heatmap comparing BSH-producing bacteria and physiological traits; (**B**) correlation heatmap comparing BSH-producing bacteria and bile acids; (**C**–**H**) relative abundances of BSH-producing bacteria. Values are represented as mean ± SD. n = 8. * *p* < 0.05, ** *p* < 0.01, and *** *p* < 0.001 vs. DC. n = 8.

Figure 6A shows that *Bacillus* and *Bifidobacterium* were positively related to FBG (*p* < 0.05) and TC (*p* < 0.01). The abundance of *Bacillus* and *Bifidobacterium* was higher in the DC mice compared to the NC mice (*p* < 0.05), and the F2-L and F2-H interventions decreased their abundance (*p* < 0.05, Figure 6C,D). Moreover, *Lactococcus* was also found to be positively related to FBG, and *Lactococcus* was barely present in the F2 group. Considering all of the findings, BSH-producing bacteria may be the therapy targets of F2, including *Bacillus*, *Bifidobacterium*, and *Lactococcus*.

### 3.5. Correlations Between Bile Acids and BSH-Producing Bacteria

To explore the relationships between bile acids and BSH-producing bacteria, a Spearman analysis was performed (Figure 6B). From the correlation heatmap, it was observed that *Lactococcus* was positively related to TCA and TCDCA and negatively related to LCA.

## 4. Discussion

Pre-DM is the transition period before T2DM, characterized by glucose and lipid disorders [5]. In this research, we found that *G. frondosa* polysaccharide F2 ameliorated lipid and glucose metabolism disorders in pre-DM mice.

The metabolites in feces play important roles as signaling mediators, immune regulators, environmental sensors, and even endogenous toxins [28]. With the development of metabolomics technology, high-throughput mapping metabolites as profiles has become possible, which is useful for screening potential biomarkers of pathogenesis [29] in feces and exploring the therapeutic features and clinical efficacy of drugs and candidates [30]. In the present study, the pathway analysis of metabolomics showed that bile acid metabolism disorders occurred in pre-DM mice.

Bile acids are present in humans as metabolites for modulating lipid, glucose, and energy metabolism and maintaining metabolic homeostasis through bile-acid-related signaling pathways [31,32]. Following the intervention of polysaccharide F2 (60 mg/kg/d, 5 w), F2 reduced the levels of conjugated bile acids, including TCDCA and TCA, and increased the levels of free bile acids, such as LCA. Free bile acids, because of their low solubility, are easily excreted from the gall bladder into the intestine, reducing the pool of bile acids and thereby increasing the utilization of serum cholesterol by hepatocytes to synthesize bile acids. It could be speculated that F2 may promote the conversion of conjugated bile acids (such as TCDCA and TCA) to free bile acids (such as LCA) to increase the excretion of bile acids, thereby promoting further catabolism of cholesterol to function for alleviating glucose and lipid metabolism.

BSH is indispensable in the bile-acid-mediated signaling pathway [33]. Free bile acids, conjugated with taurine or glycine, enter the intestine through gallbladder contractions and are then deconjugated with BSH produced by bacteria [34]. The gut microbiota, especially BSH-producing bacteria, plays an important role in bile acid metabolism by regulating the ratio of free bile acids to bound bile acids, thereby improving host glucose and lipid metabolism [35]. *Lactobacillus*, *Lactococcus*, *Streptococcus*, *Bacillus*, *Enterococcus*, *Bifidobacterium*, *Bacteroides*, and *Bacteroides fragilis* were reported as BSH-producing bacteria [34,36]. The correlation heatmap results between bile acids and BSH-producing bacteria confirmed the connection between *Lactococcus* and TCA/TCDCA/LCA, which suggested that F2 may ameliorate lipid and glucose metabolism through the regulation of the *Lactococcus*–TCA/TCDCA and *Lactococcus*–LCA axes. Therefore, it may be speculated that F2 may ameliorate glucose and lipid metabolism disorders by promoting bile acid metabolism and regulating the abundance of BSH-producing bacteria (*Lactococcus* spp.).

Under normal conditions, the synthesis of 75% of bile acids is initiated by the 7a-hydroxylation of cholesterol, which is catalyzed by the rate-limiting enzyme cholesterol 7a-hydroxylase (CYP7A1) in the liver. It has been reported that TCA in the intestine may inhibit the transcription of CYP7A1 by promoting the farnesoid X receptor (FXR)–fibroblast growth factor (FGF)15/FGF19–FGFR4 pathway and that TCDCA inhibits CYP7A1 transcription by activating the FXR/small heterodimer partner (SHP) pathway in the liver [34,37,38]. In the present study, the decrease in TCA/TCDCA concentration caused by F2 intervention may have weakened the inhibitory effect of CYP7A1 and promoted the conversion of cholesterol to bile acids in the liver.

LCA is the activator of TGR5, which can promote the secretion of glucagon-like peptide-1 (GLP-1) by activating downstream cAMP/PKA, improve insulin resistance, and regulate host glucose metabolism [39,40]. In the present study, F2-H intervention significantly increased the level of LCA, which may modulate the TGR5 pathway.

**Figure 7:**
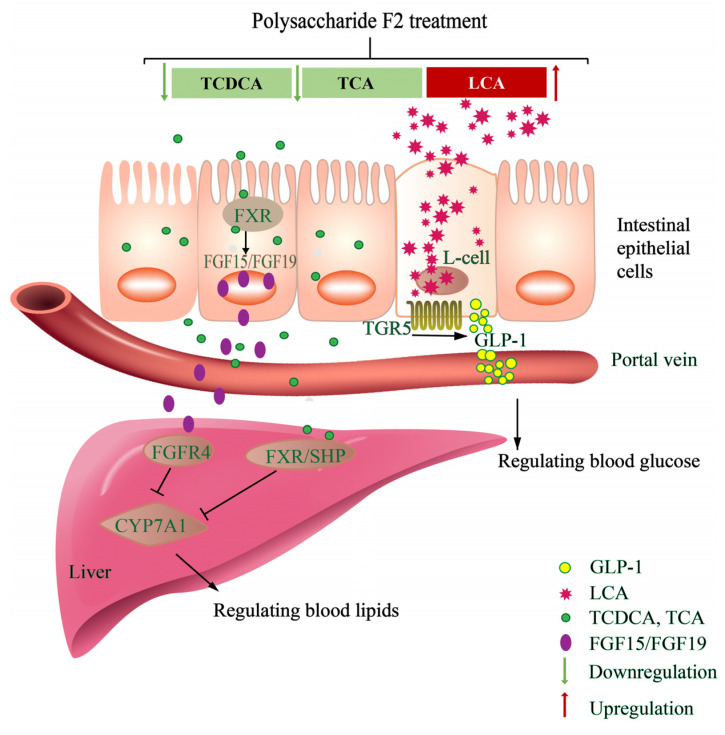
A proposed pathway for the effect of F2 on glucose and lipid metabolism. Red represents increase and green represents decrease.

Therefore, it is believed that F2 may increase the level of LCA, contributing to the modulation of the TGR5 pathway and decreasing blood glucose, while simultaneously decreasing TCDCA and TCA, thus promoting liver lipid metabolism, ultimately achieving the effect of regulating glucose and lipid metabolism (Figure 7).

However, sex-specific differences in lipid and glucose metabolism should not be ignored [41]. These differences seem to be due to higher whole-body insulin sensitivity in females; however, the mechanisms responsible for sex differences in insulin sensitivity are not yet understood [42]. In the present study on the regulatory effects of GF5000 on blood lipids and glucose, the inclusion of only male mice in animal experiments is not comprehensive and is a limitation of this study. In addition, the key metabolites, intestinal bacteria, and their interactions in *G. frondosa* polysaccharide F2 in improving glucose and lipid metabolism in pre-DM mice would be validated further through fecal microbiota transplantation experiments.

For functional foods, safety is the most important factor, so it is urgent to carry out acute and long-term safety evaluations of *G. frondosa* polysaccharide F2. On this basis, small-scale human clinical trials will be conducted to provide a theoretical basis for the development of functional foods.

## 5. Conclusions

In this research, we reported the amelioration of lipid and glucose metabolism disorders by *G. frondosa* polysaccharide F2 through the modulation of bile acids in pre-DM mice. The untargeted metabolomics results showed that F2 regulated bile acid metabolism by reducing the levels of conjugated bile acids, including TCDCA and TCA, and increasing the levels of free bile acids, such as LCA. The 16S rDNA sequencing results showed that BSH-producing bacteria may be the therapy targets of F2, including *Bacillus*, *Bifidobacterium*, and *Lactococcus*. Through the integrated analysis of untargeted metabolomics and 16S rDNA sequencing data, we explored the mechanism of improvement of glucose and lipid metabolism disorders in pre-DM mice, which may be regulated through the Lactococcus–TCA/TCDCA–FXR–CYP7A1 and *Lactococcus*–LCA–TGR5–GLP-1 axes. This research may provide novel insights into the crosstalk between bile acids and gut microbiota in the intervention of pre-DM by *G. frondosa* polysaccharide F2.

## Figures and Tables

**Table 1:** F2 decreased FBG of pre-DM mice within 5 weeks.

Group	Fasting Blood Glucose (FBG, mmol/L)				
	Week 1	Week 2	Week 3	Week 4	Week 5
NC	7.27 ± 1.00 **	6.61 ± 0.71 *	6.88 ± 0.64	6.49 ± 0.71 **	6.13 ± 0.61 ****
DC	8.33 ± 0.62	7.35 ± 0.54	6.67 ± 0.73	7.27 ± 0.77	7.34 ± 0.67
PC	7.86 ± 0.63	6.84 ± 0.58	6.69 ± 0.78	6.67 ± 0.55 *	6.42 ± 0.8 **
F2-L	7.77 ± 0.96	6.20 ± 1.11 **	6.53 ± 0.74	6.49 ± 0.67 **	6.12 ± 0.55 ****
F2-H	7.80 ± 0.55	6.98 ± 0.97	6.38 ± 0.99	6.62 ± 0.45 *	6.37 ± 0.57 **

Values represent means ± SD (n = 8). * *p* < 0.05, ** *p* < 0.01, **** *p* < 0.0001 vs. DC.

**Table 2:** F2 ameliorated IGT of pre-DM mice in OGTT.

Group	Blood Glucose (mmol/L)				
	0 h	0.5 h	1 h	1.5 h	2 h
NC	4.00 ±0.41 ****	13.25 ± 1.98	7.13 ± 1.00	5.07 ± 0.36 ****	4.58 ± 0.39 ****
DC	5.53 ± 0.64	12.24 ± 2.17	8.51 ± 1.25	6.77 ± 1.43	5.94 ± 0.89
PC	3.44 ± 0.40 ****	11.37 ± 3.26	8.00 ± 2.09	5.54 ± 0.84 **	4.83 ± 0.71 ****
F2-L	4.81 ± 0.62 **	13.27 ± 2.43	8.73 ± 1.24	6.31 ± 0.65	5.51 ± 0.70
F2-H	4.87 ± 0.67 **	15.46 ± 3.65 *	9.91 ± 2.19	6.28 ± 0.70	5.73 ± 0.65

n = 8. Values represent means ± SD. * *p* < 0.05, ** *p* < 0.01, **** *p* < 0.0001 vs. DC.

## Data Availability

The original contributions presented in the study are included in the article/Supplementary Materials, further inquiries can be directed to the corresponding authors.

## Supplementary Materials

The following supporting information can be downloaded at https://www.mdpi.com/article/10.3390/foods14060955/s1: Table S1: The composition of high-fat and high-sugar (HFHS) diet for prediabetic model establishment; Table S2: Different expressed metabolites (DEMs) in the comparison of pre-DM control (DC) vs. normal control (NC); Table S3: Characteristic metabolism pathways in the comparison of DC vs. NC; Table S4: Different expressed metabolites (DEMs) in the comparison of F2 group at a high dose (F2-H) vs. DC; Table S5: Characteristic metabolism pathways in the comparison of F2-H vs. DC.
