# Supplementary material for: Grifola frondosa Polysaccharide F2 Ameliorates Disordered Glucose and Lipid Metabolism in Prediabetic Mice by Modulating Bile Acids
Source: Foods. 2025 Mar 11;14(6):955. doi: 10.3390/foods14060955 (PMC11941230; doi:10.3390/foods14060955)
Supplement: Supplementary file 1 [file foods-14-00955-s001.zip › foods-3373142-supplementary.pdf]

Supplementary Materials

Grifola frondosa Polysaccharide F2 Ameliorates Disordered Glucose and Lipid Metabolism in Prediabetic Mice By Modu-lating Bile Acids

Ruifang Zhang[1, †], Chun Xiao[1, †], Tianqiao Yong[1, †], Longhua Huang[1], Huiping Hu [1],

Yizhen Xie[1, 2 *], Qingping Wu[1, *]

[1] Guangdong Academy of Science Institute of Microbiology, Guangdong Provincial Key Laboratory of Microbial Safety and Health, State Key Laboratory of Applied Microbiology Southern China, National Health Commission Science and Technology Innovation Platform for Nutrition and Safety of Microbial Food, Guangzhou 510070, P.R. China

[2] Guangdong Yuewei Edible Fungi Technology Co., Ltd., Guangzhou 510663, P.R. China

1181281653@qq.com (R.Z.); xiaochun960@hotmail.com (C.X.); tianqiao@mail.ustc.edu.cn (T.Y.); hlh1030@126.com (L.H.); hhp201@126.com (H.H.)

[*]Correspondence: xieyizhen@126.com (Y.X.); wuqp203@163.com (Q.W.); Tel.: +86-20-8768-8132 (Y.X.); +86-20-8768-8132 (Q.W.)

[†] These authors contributed equally to this work.

Table S1 The composition of high fat and high sugar (HFHS) diet for prediabetic model establishment.

High fat and high sugar diet	
**Ingredients**	**Percentage (%)**
Crude protein .	17.5
Crude fat	17.9
Crude fiber	3.1
Crude ash content	4.5
Moisture	8.5
Calcium	0.88
Total Phosphorus	0.58
Nitrogen-free extract	48.5

Table S2 Different expressed metabolites (DEMs) in the comparison of pre-DM control (DC) vs normal control (NC).

**Metabolites**	**HMDB ID**	***P*-value**	**Fold change (NC/DC)**	**VIP**
2-Diethylaminoethanol	HMDB0033971	2.69E-09	0.19	1.29
Methacholine	HMDB0015654	5.35E-04	2.38	5.59
Docosahexaenoyl Ethanolamide	HMDB0013658	1.20E-03	0.37	1.33
Stearoylethanolamide	HMDB0013078	6.96E-03	0.42	3.25
Alpha-Linolenoyl ethanolamide	HMDB0013624	1.78E-05	3.26	1.01
Oleoylethanolamide	HMDB0002088	1.96E-04	0.39	1.43
1,3,7,12-Tetrahydroxycholan-24-oic acid	HMDB0000433	3.38E-04	0.25	1.46
Deoxycholic acid	HMDB0000626	1.58E-03	0.12	7.92
Glycocholic acid	HMDB0000138	3.98E-04	0.17	7.31
Lithocholic acid	HMDB0000761	5.02E-03	0.39	2.57
3a,6b,7b,12b-Tetrahydroxy-5b-cholanoic acid	HMDB0000377	2.52E-05	0.33	1.21
Cholic acid	HMDB0000619	3.05E-05	0.22	2.05
alpha-Tocopherolquinone	HMDB0034408	7.44E-03	2.05	2.23
MG(0:0/20:4(5Z,8Z,11Z,14Z)/0:0)	HMDB0004666	2.62E-03	0.38	1.45
LysoPA(18:0/0:0)	HMDB0007854	2.63E-03	3.67	1.03
LysoPA(O-18:0/0:0)	HMDB0011144	3.44E-04	3.13	2.20
LysoPE(14:0/0:0)	HMDB0011500	4.55E-02	2.61	1.22
Diferuloylputrescine	HMDB0033468	3.68E-03	4.38	2.86
Equol	HMDB0002209	2.91E-03	0.38	1.08
Jasmonic acid	HMDB0032797	1.30E-04	2.28	1.52
DG(15:0/18:3(6Z,9Z,12Z)/0:0)	HMDB0007075	3.22E-06	4.80	1.19
PGF2a ethanolamide	HMDB0013628	2.37E-05	2.06	1.06
Taurine	HMDB0000251	3.31E-02	0.34	1.30
N-Carboxyethyl-g-aminobutyric acid	HMDB0002201	3.36E-06	3.30	1.05
Diaminopimelic acid	HMDB0001370	5.56E-03	3.06	2.70
N-Acetyl-L-alanine	HMDB0000766	4.52E-06	2.00	1.04
N-Lauroylglycine	HMDB0013272	1.37E-04	2.25	2.77
Pipecolic acid	HMDB0000070	1.95E-03	6.22	10.96
cis-5-Tetradecenoylcarnitine	HMDB0002014	6.52E-04	3.78	2.08
Choline	HMDB0000097	5.72E-03	2.16	3.27
Annocherin A	HMDB0033399	3.86E-04	0.37	1.47
LysoPC(18:3(9Z,12Z,15Z))	HMDB0010388	2.70E-03	2.83	1.83
LysoPC(18:2(9Z,12Z)/0:0)	HMDB0010386	7.36E-06	2.93	10.28
LysoPC(14:0/0:0)	HMDB0010379	3.79E-04	3.26	2.17

continued-

Metabolites	HMDB ID	P-value	FC	VIP
LysoPC(20:4(5Z,8Z,11Z,14Z)/0:0)	HMDB0010395	3.34E-04	0.39	1.28
LysoPE(P-16:0/0:0)	HMDB0011152	2.93E-04	3.76	5.37
LysoPE(0:0/18:2(9Z,12Z))	HMDB0011477	3.04E-05	2.44	1.45
3-Methylcyclopentadecanone	HMDB0034181	5.67E-07	0.23	1.03
Alpha-Linolenic acid	HMDB0001388	1.26E-06	4.06	2.25
MG(18:2(9Z,12Z)/0:0/0:0)	HMDB0011568	1.93E-04	3.07	8.80
Linoleic acid	HMDB0000673	7.15E-05	2.01	21.27
16-Hydroxy 4exadecenoic acid	HMDB0006294	1.58E-03	2.23	1.14
Heptadecanoic acid	HMDB0002259	2.77E-04	0.40	1.01
Myristoleic acid	HMDB0002000	5.85E-08	0.18	1.91
Palmitoleic acid	HMDB0003229	3.05E-08	0.30	8.63
Docosapentaenoic acid (22n-6)	HMDB0001976	6.76E-06	0.36	4.13
Hypogeic acid	HMDB0002186	2.06E-07	0.24	8.93
Floionolic acid	HMDB0034295	1.17E-05	2.60	1.48
Ricinoleic acid	HMDB0034297	1.47E-03	2.86	1.17
Adrenic acid	HMDB0002226	6.76E-06	0.36	4.13
Eicosapentaenoic acid	HMDB0001999	1.13E-05	0.44	2.65
Docosapentaenoic acid	HMDB0006528	1.17E-03	0.44	1.15
Avocadene 2-acetate	HMDB0031044	8.40E-06	0.18	2.32
Exaltolide	HMDB0034455	1.83E-03	0.42	1.09
MG(18:0/0:0/0:0)	HMDB0011131	4.48E-06	0.13	2.94
5-Hydroxyindolepyruvate	METPA0633	3.99E-02	3.13	1.58
Carbamazepine	HMDB0014704	3.67E-04	0.30	1.45
4-Imidazolone-5-propionic acid	HMDB0001014	2.15E-04	2.12	1.04
Palmitoylethanolamide	HMDB0002100	8.39E-03	0.37	5.37
MG(16:0/0:0/0:0)	HMDB0011564	5.28E-06	0.18	5.65
Phendimetrazine	HMDB0015519	2.50E-06	0.11	1.13
Thiamine	HMDB0000235	2.11E-03	0.30	2.55
Uracil	HMDB0000300	2.92E-03	2.01	2.71
Mesobilirubinogen	HMDB0001898	3.68E-03	0.16	1.62
Xanthine	HMDB0000292	8.48E-04	3.85	1.65
6-Methylquinoline	HMDB0033115	5.21E-03	0.15	2.78
Anacardic acid	HMDB0033896	2.04E-04	2.39	1.05
(1E)-1-(methylthio)prop-1-ene	HMDB0059843	8.90E-05	0.06	1.15
Momordicinin	HMDB0034726	4.92E-04	0.49	1.22

Table S3 Characteristic metabolism pathways in the comparison of DC *vs* NC.

Pathway name	Total	Hits	p	FDR	Impact
α-Linolenic acid metabolism	13	1	1.26E-06	2.02E-05	0.33333
Biosynthesis of unsaturated Fatty acids	36	3	7.14E-05	0.000382	0
Linoleic acid metabolism	5	1	7.15E-05	0.000382	1
Histidine metabolism	16	1	0.000215	0.000859	0.09016
Primary bile acid biosynthesis	46	3	0.000392	0.001254	0.04524
Purine metabolism	66	1	0.000848	0.002261	0.02966
Lysine degradation	25	1	0.001949	0.003598	0
Thiamine metabolism	7	1	0.00211	0.003598	0
Glycerolipid metabolism	16	1	0.002635	0.003598	0.01246
Phosphatidylinositol signaling system	28	1	0.002635	0.003598	0.00152
Pyrimidine metabolism	39	1	0.002924	0.003598	0.0743
beta-Alanine metabolism	21	1	0.002924	0.003598	0
Pantothenate and CoA biosynthesis	19	1	0.002924	0.003598	0
Glycerophospholipid metabolism	36	3	0.004028	0.004603	0.18305
Glycine, serine and threonine metabolism	34	1	0.00572	0.006102	0
Taurine and hypotaurine metabolism	8	1	0.033099	0.033099	0.42857

Table S4 Different expressed metabolites (DEMs) in the comparison of F2 group at a high dose (F2-H) *vs* DC.

**Metabolites**	HMDB ID	*P*-value	FC (F2H/DC)	VIP
Leucyl-Leucine	HMDB0028933	7.51E-03	3.83	1.02
Leucyl-Valine	HMDB0028942	1.45E-02	5.13	1.11
6-Hydroxyhexanoic acid	HMDB0012843	7.91E-03	2.97	1.22
Dhelwangin	HMDB0030703	1.80E-03	2.11	1.50
Benzenebutanoic acid	HMDB0000543	1.71E-02	0.46	1.34
Beta-Alanine	HMDB0000056	3.55E-03	2.28	2.57
Taurine	HMDB0000251	3.58E-02	0.33	2.24
3b-Hydroxy-5-cholenoic acid	HMDB0000308	9.98E-03	3.79	1.09
Taurochenodesoxycholic acid	HMDB0000951	1.98E-03	0.29	1.89
Taurocholic acid	HMDB0000036	3.14E-02	0.25	3.23
Mesobilirubinogen	HMDB0001898	4.75E-02	2.85	1.52
N8-Acetylspermidine	HMDB0002189	2.67E-03	3.25	1.40
cis-5-Tetradecenoylcarnitine	HMDB0002014	1.81E-03	0.38	1.36
Paucine	HMDB0029876	4.85E-03	15.70	1.14
Tetradec-2-enal	HMDB0032525	4.92E-02	0.39	1.56
N-Acetylglutamic acid	HMDB0001138	4.46E-03	2.01	1.51
MG(0:0/15:0/0:0)	HMDB0011532	6.57E-03	3.27	2.09
LysoPC(16:1(9Z))	HMDB0010383	4.34E-02	3.44	1.59
LysoPE(0:0/20:3(5Z,8Z,11Z))	HMDB0011485	3.64E-03	2.26	1.20
Sulfolithocholylglycine	HMDB0002639	3.63E-02	0.32	1.52
trans-Ferulic acid	HMDB0000954	1.21E-02	0.39	1.15
D-Tryptophan	HMDB0013609	4.31E-02	0.44	3.53
Neocnidilide	HMDB0034450	2.27E-02	0.40	4.26
Daidzein	HMDB0003312	8.93E-03	0.47	1.15
16-Hydroxy 6exadecenoic acid	HMDB0006294	6.45E-04	4.40	1.69
Palmitoleic acid	HMDB0003229	1.84E-02	7.39	1.10
Ricinoleic acid	HMDB0034297	1.12E-02	2.76	1.50
Stearic acid	HMDB0000827	1.94E-02	0.25	1.48
Floionolic acid	HMDB0034295	1.15E-02	2.21	3.39
Tiglic acid	HMDB0001470	3.38E-02	0.41	3.07
Medrysone	HMDB0014398	1.19E-02	3.23	1.04
5a-Pregnane-3,20-dione	HMDB0003759	9.18E-05	0.21	1.04
Uracil	HMDB0000300	8.57E-03	2.30	4.74
Xanthine	HMDB0000292	1.46E-02	3.05	2.55
Deoxyuridine	HMDB0000012	1.79E-02	2.49	1.06
Soyasaponin I	HMDB0034649	3.18E-02	0.49	1.87
4,4-Dimethyl-5a-cholesta-8,24-dien-3-b-ol	HMDB0001286	2.69E-03	2.42	1.19

Table S5 Characteristic metabolism pathways in the comparison of F2-H *vs* DC.

Pathway name	Total	Hits	p	FDR	Impact
Steroid hormone biosynthesis	77	1	9.18E-05	0.0011014	0.00653
Steroid biosynthesis	42	1	0.0026852	0.0133	0.08184
Propanoate metabolism	23	1	0.0035476	0.0133	0
Arginine biosynthesis	14	1	0.0044574	0.0133	0
Pyrimidine metabolism	39	3	0.0077318	0.0133	0.13159
beta-Alanine metabolism	21	2	0.0077584	0.0133	0.39925
Pantothenate and CoA biosynthesis	19	2	0.0077584	0.0133	0.02143
Purine metabolism	66	1	0.014615	0.021923	0.02966
Biosynthesis of unsaturated fatty acids	36	1	0.019421	0.025894	0
Glycerophospholipid metabolism	36	1	0.043409	0.043409	0.01736
Primary bile acid biosynthesis	46	3	0.03185	0.036834	0.06809
Taurine and hypotaurine metabolism	8	2	0.033764	0.036834	0.42857
